# Supplementary figures and images for: Lung ultrasound is associated with distinct clinical phenotypes in COVID-19 ARDS: A retrospective observational study
Source: PLoS One. 2024 Jun 3;19(6):e0304508. doi: 10.1371/journal.pone.0304508 (PMC11146726; doi:10.1371/journal.pone.0304508)

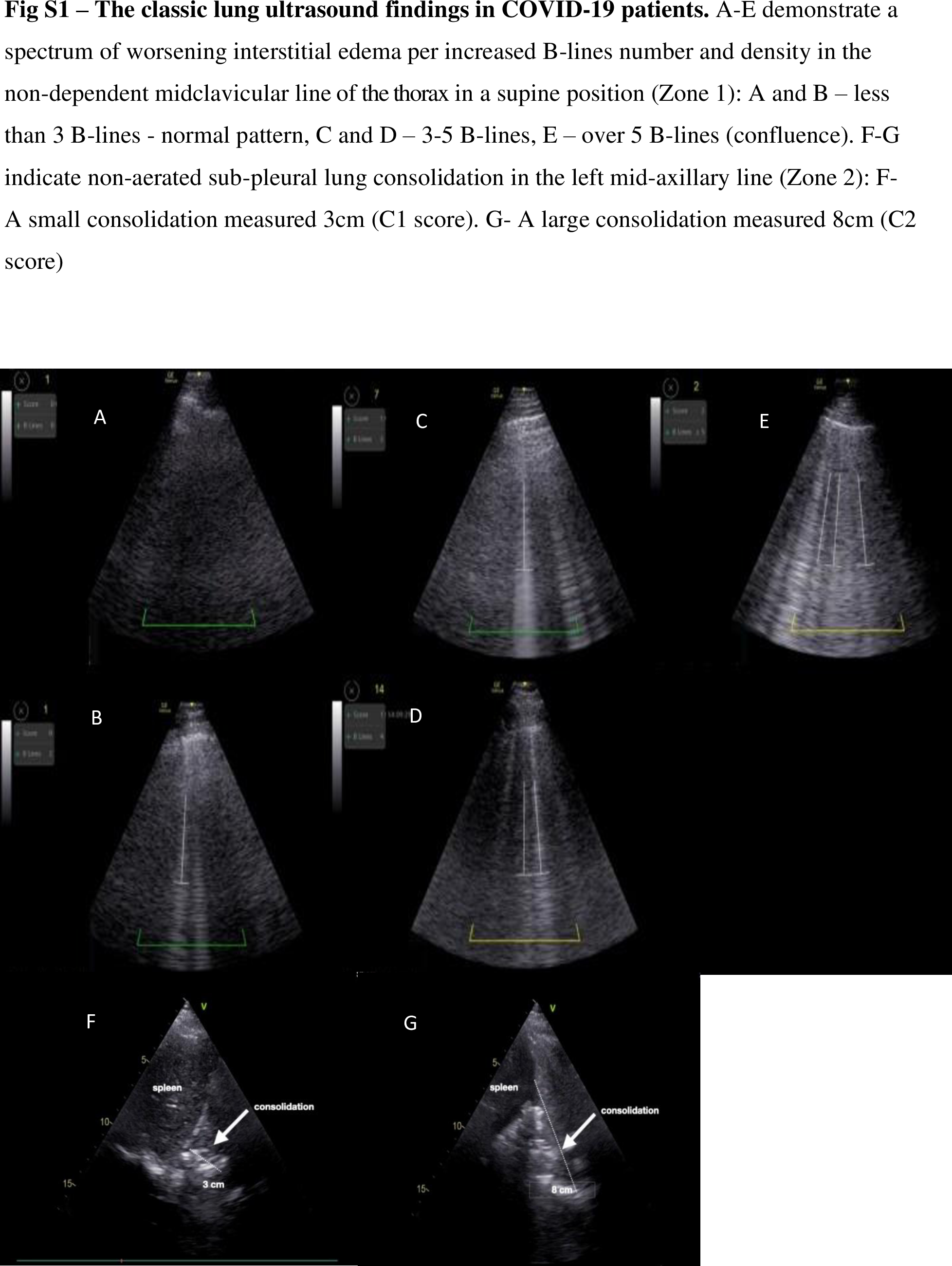

Supplement: S1 Fig — (TIF) [file pone.0304508.s001.tif]

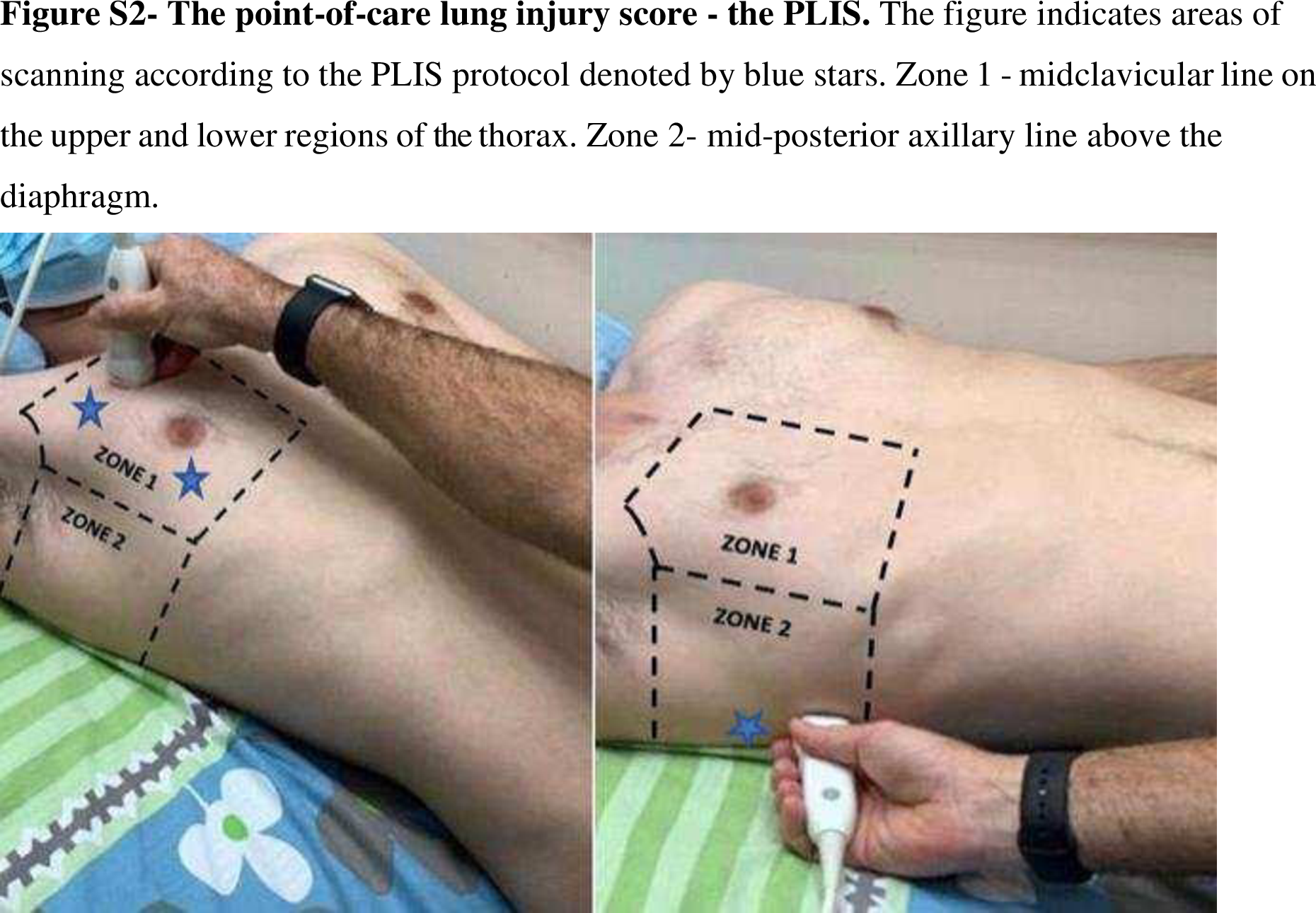

Supplement: S2 Fig — (TIF) [file pone.0304508.s002.tif]
