# Supplementary material for: Lung ultrasound is associated with distinct clinical phenotypes in COVID-19 ARDS: A retrospective observational study
Source: PLoS One. 2024 Jun 3;19(6):e0304508. doi: 10.1371/journal.pone.0304508 (PMC11146726; doi:10.1371/journal.pone.0304508)
Supplement: S1 Table — (DOCX) [file pone.0304508.s003.docx]

**S1 Table - The point of care lung ultrasound injury score (PLIS) grading system.**

| SCORE | 0 | 1 | 2 |
| --- | --- | --- | --- |
| A | Room air/nasal prongs | Any non-invasive support over nasal prongs | Intubated |
| B*  Zone 1 only –  upper and lower areas | B-lines <3 | B-lines 3-5 | B-lines > 5 |
| C**  Zone 1 - upper and lower areas  Zone 2- lower | No consolidation | Small consolidation (either unilateral or bilateral) | Large*** consolidation (either unilateral or Bilateral) |

* B-Lines contribute to the score only if located bilaterally in Zone 1. The higher number of B-lines from any side defined the grading of the B component.
** If the consolidation/s are unilateral – the C-score is attached with the letter "R"- for consolidation located only in the right lung, or "L" when they appear only in the left lung.

***Large consolidation – measurement of over 4 cm of the largest diameter.
