# Supplementary material for: Lung ultrasound is associated with distinct clinical phenotypes in COVID-19 ARDS: A retrospective observational study
Source: PLoS One. 2024 Jun 3;19(6):e0304508. doi: 10.1371/journal.pone.0304508 (PMC11146726; doi:10.1371/journal.pone.0304508)
Supplement: S2 Table — (DOCX) [file pone.0304508.s004.docx]

# S2 Table: Comprehensive Cohort Outcomes

| **Patient**  **ID** | **The corresponding patient number in Graphical Figure 1**  **(if applicable)** | **Age**  **(years)** | **Invasive mechanical Ventilation Day of PLIS scan** | **C-type phenotype**  **(yes/no)** | **High PEEP  (PEEP ≥ 12)**  **(yes/no)** | **Prone Position**  **(yes/no)** | **High iNO  (NO ≥ 15 ppm)**  **(yes/no)** | **PaO2/FiO2** | **Survival to discharge (yes/no)** |
| --- | --- | --- | --- | --- | --- | --- | --- | --- | --- |
| 01 | 1 | 56 | 1 | Yes | No | No | No | 124 | Yes |
|  |  |  | 3 | Yes | No | No | No | 145 |  |
|  |  |  | 6 | No | No | No | No | 255 |  |
|  |  |  | 7 | Yes | No | No | No | 343 |  |
| 02 | 2 | 57 | 1 | No | Yes | No | No | 199 | Yes |
|  |  |  | 2 | Yes | No | No | Yes | 176 |  |
|  |  |  | 3 | Yes | Yes | No | Yes | 191 |  |
|  |  |  | 4 | Yes | Yes | No | No | 121 |  |
|  |  |  | 5 | Yes | Yes | No | Yes | 101 |  |
|  |  |  | 7 | Yes | Yes | Yes | Yes | 177 |  |
|  |  |  | 8 | Yes | Yes | Yes | Yes | 99 |  |
|  |  |  | 9 | Yes | No | Yes | No | 95 |  |
|  |  |  | 11 | Yes | Yes | Yes | Yes | 179 |  |
|  |  |  | 12 | Yes | Yes | No | Yes | 219 |  |
|  |  |  | 13 | No | Yes | No | No | 286 |  |
|  |  |  | 14 | Yes | Yes | No | No | 276 |  |
|  |  |  | 15 | Yes | No | No | No | 318 |  |
|  |  |  | 16 | No | No | No | No | 232 |  |
|  |  |  | 17 | Yes | No | No | No | 250 |  |
|  |  |  | 18 | No | No | No | No | 245 |  |
|  |  |  | 19 | No | No | No | No | 205 |  |
|  |  |  | 20 | No | No | No | No | 208 |  |
|  |  |  | 21 | No | No | No | No | 235 |  |
|  |  |  | 22 | Yes | No | No | No | 295 |  |
|  |  |  | 23 | Yes | No | No | No | 290 |  |
|  |  |  | 24 | Yes | No | No | No | 360 |  |
|  |  |  | 25 | Yes | No | No | No | 298 |  |
|  |  |  | 26 | Yes | No | No | No | 80 |  |
|  |  |  | 27 | No | No | No | No | 78 |  |
|  |  |  | 29 | Yes | NA | No | No | 45 |  |
|  |  |  | 30 | No | NA | No | No | NA |  |
| 03 | 3 | 67 | 1 | Yes | Yes | No | No | 75 | Yes |
|  |  |  | 2 | Yes | Yes | No | Yes | 174 |  |
|  |  |  | 3 | Yes | Yes | No | No | 144 |  |
|  |  |  | 4 | Yes | Yes | No | No | 172 |  |
|  |  |  | 5 | Yes | No | No | No | 190 |  |
|  |  |  | 6 | Yes | No | No | No | 180 |  |
|  |  |  | 7 | Yes | No | No | No | 180 |  |
|  |  |  | 8 | Yes | No | No | No | 213 |  |
|  |  |  | 9 | No | No | No | No | 210 |  |
|  |  |  | 10 | No | No | No | No | 235 |  |
| 04 | 4 | 71 | 1 | No | Yes | No | No | 283 | Yes |
|  |  |  | 2 | Yes | Yes | No | No | 192 |  |
|  |  |  | 3 | No | Yes | No | No | 107 |  |
|  |  |  | 4 | Yes | Yes | No | No | 73 |  |
|  |  |  | 5 | Yes | No | No | No | 91 |  |
|  |  |  | 6 | Yes | Yes | No | No | 136 |  |
|  |  |  | 9 | No | Yes | Yes | Yes | 84 |  |
|  |  |  | 10 | No | Yes | Yes | Yes | 136 |  |
|  |  |  | 11 | Yes | Yes | No | Yes | 163 |  |
|  |  |  | 12 | Yes | Yes | No | No | 137 |  |
|  |  |  | 13 | Yes | Yes | No | No | 108 |  |
|  |  |  | 14 | Yes | Yes | No | No | 129 |  |
|  |  |  | 15 | Yes | No | No | No | 138 |  |
|  |  |  | 16 | No | No | No | No | 166 |  |
|  |  |  | 17 | No | No | No | No | 136 |  |
|  |  |  | 18 | No | No | No | No | 178 |  |
|  |  |  | 19 | No | No | No | No | 328 |  |
| 05 | 5 | 52 | 2 | Yes | No | No | No | 185 | Yes |
|  |  |  | 3 | No | No | No | No | 189 |  |
|  |  |  | 7 | Yes | No | No | No | 250 |  |
|  |  |  | 8 | Yes | No | No | No | 328 |  |
| 06 | NA | 63 | 1 | Yes | No | No | No | 753 | Yes |
| 07 | NA | 72 | 11 | No | No | No | No | 429 |  |
|  |  |  | 17 | No | No | No | No | 238 |  |
|  |  |  | 18 | No | No | No | No | 240 |  |
|  |  |  | 20 | No | No | No | No | 88 |  |
|  |  |  | 25 | No | No | No | No | 140 |  |
| 08 | NA | 60 | 1 | No | Yes | No | No | 468 | Yes |
| 09 | 6 | 58 | 4 | Yes | No | No | No | 136 | No |
|  |  |  | 6 | Yes | No | No | No | NA |  |
|  |  |  | 10 | No | No | No | No | 308 |  |
|  |  |  | 11 | Yes | No | No | No | 116 |  |
|  |  |  | 13 | Yes | Yes | Yes | No | 188 |  |
|  |  |  | 14 | Yes | Yes | Yes | No | 83 |  |
|  |  |  | 17 | Yes | Yes | No | No | 188 |  |
|  |  |  | 18 | Yes | Yes | No | No | 199 |  |
|  |  |  | 20 | Yes | Yes | No | No | 195 |  |
|  |  |  | 21 | Yes | Yes | No | No | 65 |  |
| 10 | 7 | 69 | 1 | Yes | NA | No | No | NA | No |
|  |  |  | 6 | No | No | No | No | 290 |  |
|  |  |  | 7 | Yes | No | No | No | 234 |  |
|  |  |  | 9 | Yes | No | No | No | 295 |  |
|  |  |  | 14 | Yes | No | Yes | No | 95 |  |
|  |  |  | 17 | Yes | No | No | No | 178 |  |
|  |  |  | 18 | Yes | No | No | No | 116 |  |
|  |  |  | 35 | Yes | No | No | Yes | 114 |  |
|  |  |  | 45 | Yes | No | No | Yes | 54 |  |
| 11 | 8 | 36 | 3 | No | Yes | No | No | 535 | No |
|  |  |  | 4 | No | Yes | No | No | 125 |  |
|  |  |  | 5 | Yes | Yes | No | No | 176 |  |
|  |  |  | 7 | Yes | Yes | No | No | 290 |  |
|  |  |  | 9 | Yes | Yes | No | No | 130 |  |
|  |  |  | 26 | No | Yes | No | Yes | 57 |  |
| 12 | 9 | 64 | 1 | No | No | No | Yes | 128 | No |
|  |  |  | 2 | No | Yes | No | No | 124 |  |
|  |  |  | 3 | Yes | Yes | No | No | 76 |  |
|  |  |  | 4 | Yes | Yes | Yes | Yes | 92 |  |
|  |  |  | 5 | Yes | Yes | Yes | Yes | 88 |  |
|  |  |  | 6 | Yes | Yes | Yes | Yes | 62 |  |
|  |  |  | 7 | Yes | Yes | Yes | Yes | 63 |  |
| 13 | 10 | 67 | 1 | Yes | Yes | No | Yes | 121 | No |
|  |  |  | 2 | Yes | Yes | No | No | 129 |  |
|  |  |  | 3 | Yes | Yes | Yes | Yes | 96 |  |
|  |  |  | 5 | No | Yes | Yes | Yes | 105 |  |
|  |  |  | 6 | No | Yes | Yes | Yes | 158 |  |
| 14 | NA | 82 | 1 | No | No | No | No | 58 | No |
|  |  |  | 2 | Yes | No | Yes | Yes | 78 |  |
| 15 | NA | 60 | 2 | Yes | Yes | Yes | Yes | 130 | No |
| 16 | NA | 56 | 2 | Yes | Yes | No | No | 258 |  |
|  |  |  | 3 | Yes | Yes | No | No | 116 |  |
|  |  |  | 7 | Yes | Yes | No | No | 78 |  |
|  |  |  | 9 | No | Yes | No | No | 321 |  |
| 17 | NA | 90 | 2 | No | NA | No | No | NA | No |
| 18 | NA | 54 | 2 | No | Yes | No | No | 226 | No |
| 19 | NA | 76 | 3 | No | No | No | No | 104 | No |
|  |  |  | 5 | Yes | No | No | No | 69 |  |
| 20 | NA | 83 | 2 | Yes | NA | No | No | NA | No |
|  |  |  | 3 | No | Yes | No | No | 123 |  |
|  |  |  | 4 | No | Yes | No | No | 75 |  |
|  |  |  | 5 | No | Yes | No | No | 136 |  |
|  |  |  | 11 | No | No | No | No | 104 |  |
|  |  |  | 12 | No | No | No | No | 172 |  |
| 21 | NA | 80 | 12 | Yes | NA | No | No | NA | No |
|  |  |  | 15 | Yes | Yes | No | Yes | 130 |  |
|  |  |  | 14 | Yes | Yes | No | Yes | 103 |  |
| 22 | NA | 66 | 13 | No | No | No | No | 176 | No |
| 23 | NA | 63 | 2 | Yes | Yes | No | Yes | 32 | No |

*Abbreviations: ARDS – Acute Respiratory Distress Syndrome; PEEP – Positive End-Expiratory Pressure; iNO – inhaled Nitric Oxide; PLIS – Point of Care Lung Ultrasound Injury Score; FiO2 – Fraction of Inspired Oxygen; PaO2 – Partial Pressure of Oxygen in Arterial Blood; NA – Non Applicable;* ID *– Identification Number.*
