# Supplementary material for: Lung ultrasound is associated with distinct clinical phenotypes in COVID-19 ARDS: A retrospective observational study
Source: PLoS One. 2024 Jun 3;19(6):e0304508. doi: 10.1371/journal.pone.0304508 (PMC11146726; doi:10.1371/journal.pone.0304508)
Supplement: Attachment — Submitted filename: Comments to Author.docx [file pone.0304508.s005.docx]

**Comments to Author**

**Major Comments**

-Overall, this study is well-written and organized. The Discussion in particular provides a thorough summary of existing literature and ties in the study findings nicely. I also commend the authors on performing the arduous task of performing and interpreting serial lung ultrasound examinations. However, there are major flaws with the study design that would need to be addressed.

-The author’s primary outcome is heavily flawed. The stated objective of this study was to investigate whether the presence of large consolidation on lung ultrasound alone was predictive of outcomes among COVID-19 ARDS patients. However, the primary outcome was not a measure of patient outcomes (ie. duration of mechanical ventilation, duration of ICU stay, mortality) but instead a composite of whether the patient required proning, high PEEP, or inhaled pulmonary vasodilator. The decision to employ one of these interventions is heavily biased by the treating physician and is often related to the patient’s underlying comorbidities rather than simply the severity or type of ARDS. For example, an obese patient would necessitate high PEEP regardless of the presence or severity of ARDS to facilitate alveolar recruitment and minimize atelectasis, while a patient with pulmonary hypertension may require an inhaled pulmonary vasodilator even with very mild ARDS. The combination of these 3 very different interventions into a composite outcome further weakens the study findings, as stated in the Discussion (Lines 281 – 284).

-Phenotyping in ARDS has typically referred to the underlying molecular mechanisms involved in the lung damage, based on local and systemic biomarkers, and degree of inflammation. Here, the authors instead use the term phenotype to describe whether or not the patient had large consolidations on lung ultrasound. The use of “phenotyping” in this manuscript is misleading and should be avoided or specified as “sonographic phenotyping”, as the presence of consolidation does not constitute a molecular phenotype but simply describes an imaging finding.

- In the methods, the authors state (line 139-140): " A single PLIS scan per patient composed the unit used for analysis”. This seems to be incorrect, as 128 data points (47 B-type and 81 C-type) scans were included in the results. Please clarify. If a single scan was used for each patient, please describe which scan was selected for analysis and how this selection was justified.

-The patients performed serial ultrasound examinations on each patient, which provides a robust data set when looking at each individual patient longitudinally (as shown in Figure 1) but allows for confounding if treating each scan as a separate data point, as they appear to be.

-Figure 1 is very well designed and formatted to capture the clinical courses of these patients. However, the conclusion that is drawn from this data seems contrary to the author’s main conclusion, showing that the non-survivors tended to develop consolidations while the survivors tended to have isolated B-lines without consolidation. This would imply that consolidations are associated with more severe disease, which could very well be true and this study was simply underpowered and not properly designed to detect this.

**Minor Comments**

-Lines 23, 32: please avoid or define non-standard abbreviations in the abstract, including “PLIS” and “LUS”

-Lines 31, 130: please use appropriate capitalization and subscript for “PaO_2_/FiO_2_ ratio”

-Line 42: the authors should rephrase “asymptomatic carriage”; could simply say “asymptomatic”

-Lines 44-45: I would argue against the description of COVID-19 as “ambiguous clinical presentation”. Rather, these patients routinely present with symptoms of dyspnea and increased work of breathing. Instead, I would describe how these patients often present similarly but early in their course it can be difficult to differentiate those who will progress to severe disease.

-Line 53: use distinct rather than distinctive here

-Line 54: I believe you mean restrictive fluid strategies (rather than liberal), as liberal use of fluids is not a therapeutic intervention in ARDS

-Line 66: insert comma after COVID-19. Also, this sentence needs to be shorted.

-Line 71: This should be a new sentence (after reference 18).

-Line 78: intensive care unit should not be capitalized

-Table 1: please reformat as the current formatting of data is confusing. Continuous data (mean ± SD) should be written as such. For example, age (years) should be written as 65.3 ± 11.9 rather than 65.3 (11.9). Non-continuous data should be written, for example for males, as 16 (69.9%) and specified as n (%) rather than n, %.

-Line 157: POCUS is used as an abbreviation and has not yet been defined.
