# Supplementary material for: Lung ultrasound is associated with distinct clinical phenotypes in COVID-19 ARDS: A retrospective observational study
Source: PLoS One. 2024 Jun 3;19(6):e0304508. doi: 10.1371/journal.pone.0304508 (PMC11146726; doi:10.1371/journal.pone.0304508)
Supplement: Attachment — Submitted filename: PLIS2_Revision Letter.docx [file pone.0304508.s006.docx]

Manuscript: PONE-D-23-24480

Dec 7 2023

Dear Editor in chief,

Thank you for giving us the opportunity to submit a revised draft of the manuscript “Lung ultrasound is associated with distinct clinical phenotypes in COVID-19 ARDS: A retrospective observational study” to PLOS ONE for consideration for publication. We appreciate the time and effort that you and the reviewers dedicated to providing feedback on our manuscript. We have incorporated most of the suggestions made by the reviewers. Those changes are highlighted within the manuscript.

Please see below in red for a point-by-point response to the reviewers’ comments and concerns.

All page numbers refer to the revised manuscript file, entitled “manuscript” as directed.

Funding Statement

Dr. Lior Fuchs is a consultant of General Electric healthcare. The funder has in the past previously provided support in the form of salaries for author LR but did not have any additional role in the study design, data collection and analysis, decision to publish, or preparation of the manuscript. The organization did not play any role in this research endeavor. The specific roles of authors are articulated in the ‘author contributions’ section. All co-authors have seen and agree with the contents of the manuscript, and there is no financial interest to report.

Competing Interest Statement

The above consulting relationship had no bearing or impact on the research conducted and the preparation of the manuscript. This does not alter our adherence to PLOS ONE policies on sharing data and materials. All other authors have no conflicts of interest to declare.

**REVIEWER #1**

**Point 1:** It is not clear from which point of the patient´s history the authors defined the main score of each patient. So, the final classification of each patient came from the first evaluation, maximum score, etc?
Thank you for this question. In our study the unit of analysis was the “PLIS score” and not each individual patient who met inclusion criteria. Thus, every comprehensive lung ultrasound performed served as a data point for analysis. Lung ultrasound scan studies were conducted regularly as part of morning rounds, as well as in the event of distinct clinical deterioration events and documented in the medical record. Figure 1 lays out a graphical example, where for example patient #1 had 4 PLIS scans during the period of invasive mechanical ventilation. We set out to see if a sonographic pattern based on lung ultrasound findings was associated with clinical findings at the time of the ultrasound scan. A patient’s “maximal score” or “final classification” was not pertinent to our methods or statistical analytic plan.

**Point 2:** How was the multivariable model fit? Using repeated measures? Please, clarify.
We used multivariate generalized estimating equation (GEE) regression to

analyze factors associated with the composite outcome. Using the GEE model allows for

analyzing repeated measures with non-normal response variables, such as our primary

outcome. To address this comment, the following clarification was added to the Methods

section of the revised manuscript (lines 132-134).

“The GEE model allows analyzing repeated measures with non-normal response variables, including the primary outcome.”

**Point 3:** How can a clinician know which profile a patient has? For instance, a patient score B, B, and C? or B-C-C?

Thank you for this question. If only given the overall final score, then a clinician would not be able to determine the individual components of the profile. This overall score was found to correlate with SOFA score, ICU admission, and mortality of COVID-19 pneumonia patients. This is similar to other scoring systems in medicine (e.g. SOFA). The total PLIS includes the sum of A, B, and C components. For example, the calculated PLIS for a patient on non-invasive ventilation, with more than five B- lines and small bilateral consolidations is A1B2C1 with a total PLIS of 4. The PLIS is designed to provide clinicians with information on the severity of the respiratory failure (A score) along with an image of the pathologic findings (B and C scores), thereby integrating clinical, physiologic data into a visuospatial representation of the LUS extent of affected lung. Ultimately, the total PLIS results in an overall assessment of COVID-19 severity and risk for worsening disease.

For our current study, we dichotomized the lung injury status based solely on the presence of sonographic sub-pleural consolidations. A “C-type” scanning profile was associated with higher rates of concurrently receiving a composite of high PEEP, high doses of inhaled NO, or prone positioning, regardless of illness severity. The total PLIS score was not used.

**Point 4:** This reviewer believes that the introduction is too large. It will be better to condense the introduction to provide a clear and concise overview of your study objectives. You will expand upon the detailed information in the discussion section.

Thank you for this feedback. Edits have been made appropriately. This has been incorporated into the revised manuscript**,** with a significantly edited introduction, now 525 words, decreased from 692 words in the initial version.

**REVIEWER #2**

**Point 1:** Overall, this study is well-written and organized. The Discussion in particular provides a thorough summary of existing literature and ties in the study findings nicely. I also commend the authors on performing the arduous task of performing and interpreting serial lung ultrasound examinations.

Thank you!

**Point 2:** The author’s primary outcome is heavily flawed. The stated objective of this study was to investigate whether the presence of large consolidation on lung ultrasound alone was predictive of outcomes among COVID-19 ARDS patients. However, the primary outcome was not a measure of patient outcomes (ie. duration of mechanical ventilation, duration of ICU stay, mortality) but instead a composite of whether the patient required proning, high PEEP, or inhaled pulmonary vasodilator. The decision to employ one of these interventions is heavily biased by the treating physician and is often related to the patient’s underlying comorbidities rather than simply the severity or type of ARDS. For example, an obese patient would necessitate high PEEP regardless of the presence or severity of ARDS to facilitate alveolar recruitment and minimize atelectasis, while a patient with pulmonary hypertension may require an inhaled pulmonary vasodilator even with very mild ARDS. The combination of these 3 very different interventions into a composite outcome further weakens the study findings, as stated in the Discussion (Lines 281 – 284).

Thank you for this important feedback. As mentioned by the reviewer, we explicitly acknowledge the limitation of our composite outcome in the manuscript and in our discussion. **This is addressed in lines 273-283 and the following sentence has been added**

**“**While many different clinical factors go into the decision to prone patients, initiate pulmonary vasodilators, or use a high PEEP strategy, these clinical interventions comprising the composite primary outcome are all associated with a more severe ARDS illness course. Thus, the significant association with large sonographic subpleural consolidations still serves as an important finding, despite the limitations of using a composite measurement.”

We agree with the reviewer that many different clinical factors go into the decision to initiate pulmonary vasodilators or use a high PEEP strategy. However, the common thread between the various elements of our composite outcome is that all 3 elements are associated with more severe illness and a more complicated clinical course. Thus, the finding that sonographic sub-pleural consolidations are associated with a composite of these elements has significant potential to contribute to clinical care. Our objective was to determine if the sonographic pattern based on lung ultrasound findings was associated with real world clinical findings at the time of ultrasound scan, and we believe our findings are thought-provoking and should be further studied on a larger scale with a controlled population.

The strength of lung ultrasound lies in its feasibility and simplicity and is designed to be quick and easy to perform, as one element of a comprehensive clinical evaluation. We showed that the dichotomization of lung injury status based solely on the presence of sonographic sub-pleural consolidations was associated with higher rates of concurrently receiving a composite of high PEEP, high doses of inhaled NO, or prone positioning, regardless of illness severity.

The reviewer suggests using patient outcomes such as “duration of mechanical ventilation, duration of ICU stay, mortality” - In our study the unit of analysis was the “PLIS score” and not each individual patient who met inclusion criteria, and so it would be difficult as a pilot study to analyze these outcomes.

**Point 3:** Phenotyping in ARDS has typically referred to the underlying molecular mechanisms involved in the lung damage, based on local and systemic biomarkers, and degree of inflammation. Here, the authors instead use the term phenotype to describe whether or not the patient had large consolidations on lung ultrasound. The use of “phenotyping” in this manuscript is misleading and should be avoided or specified as “sonographic phenotyping”, as the presence of consolidation does not constitute a molecular phenotype but simply describes an imaging finding.

Thank you for this comment. We have followed the guidance of the 2023 ARDS European Society of Intensive Care Medicine ARDS guidelines in our terminology (citation #35 in the manuscript). In the Guideline section dedicated exclusively to ARDS phenotyping, phenotype is established as “a clinically observable set of traits resulting from an interaction of genotype and environmental exposures” and a sub-phenotype is defined as “a distinct subgroup (of ARDS patients) that can be reliably discriminated from other subgroups based on a set or pattern of observable or measurable properties. Discrimination is typically based on a data-driven assessment of a multidimensional description of traits”

The citation for these guidelines is: Grasselli, Giacomo, et al. "ESICM guidelines on acute respiratory distress syndrome: definition, phenotyping and respiratory support strategies." *Intensive Care Medicine* 49.7 (2023): 727-759.

As such, we respectively disagree with the Reviewer, and do not think that phenotyping exclusively refers to molecular mechanisms. We believe that the radiographic/sonographic phenotyping our trial describes fits firmly within the current standard of ARDS phenotyping.

The LIVE trial (citation #10 in the manuscript) serves as a similar conceptual example, where patients were randomized to either standard lung-protective ventilation or a personalized treatment strategy based on radiological sub-phenotype. i.e. focal or diffuse pathology on chest radiograph. In the LIVE trial, a personalized PEEP and prone positioning strategy based on diffuse vs focal radiographic sub-phenotype achieved a reduction in 90-day mortality, when only considering per-protocol treated patients.

**Point 4**: In the methods, the authors state (line 139-140): " A single PLIS scan per patient composed the unit used for analysis”. This seems to be incorrect, as 128 data points (47 B-type and 81 C-type) scans were included in the results. Please clarify. If a single scan was used for each patient, please describe which scan was selected for analysis and how this selection was justified.

Thank you for this comment. We appreciate the discrepancy pointed out by the reviewer. In our study the unit of analysis was the “PLIS score” and not each individual patient who met inclusion criteria. Thus, every comprehensive lung ultrasound performed served as a data point for analysis and data was not limited to one scan per patient. The language of the manuscript has been edited to better reflect this. **Note the change on line 126-127**

**“**Each single PLIS scan obtained from the patients who met inclusion criteria composed the unit used for analysis”

**Point 5:** The patients performed serial ultrasound examinations on each patient, which provides a robust data set when looking at each individual patient longitudinally (as shown in Figure 1) but allows for confounding if treating each scan as a separate data point, as they appear to be.

Thank you for this comment. While confounding is possible, our data demonstrates that the 2 dichotomized cohorts were well balanced without significant differences. While unmeasured confounders are possible given the retrospective nature of our study, Table 2 illustrates that at the time of a given lung ultrasound evaluation for a patient, the only measurable differences were the presence of lung ultrasound consolidations and the composite primary outcome of advanced ARDS therapeutic interventions. Our findings are meant to complement and mirror real world clinical care and serve as a tool to the clinician at the bedside in the moment.

**Point 6:** Figure 1 is very well designed and formatted to capture the clinical courses of these patients. However, the conclusion that is drawn from this data seems contrary to the author’s main conclusion, showing that the non-survivors tended to develop consolidations while the survivors tended to have isolated B-lines without consolidation. This would imply that consolidations are associated with more severe disease, which could very well be true and this study was simply underpowered and not properly designed to detect this.

Thank you for this feedback. The figure graphically represents the course of ten ventilated patients with COVID-19 ARDS, or whom half survived and half died. This is meant to serve as a visual representation of our study concept and does not represent statistical findings or conclusions. As stated, patients with the most available PLIS data were selected to represent the cohort visually. Language has been added to the manuscript to ensure this is clear

Line 188: Patients with the most available PLIS data were selected **to represent the study concept.**

Line 216-218: Fig 1 graphically delineates the course of ten representative patients during their ICU **stay to illustrate the study concept. Those with the most PLIS LUS scans available were chosen for the purposes of visual representation**.

Similarly, our data did not allow for conclusions regarding survival. Our results demonstrate that for similar SOFA scores, Pao2/Fio2 ratios, and markers of clinical illness severity, those characterized by a “C-type” PLIS study with ultrasound consolidations were more likely to be treated with inhaled NO at the time of POCUS scanning and had a significantly increased likelihood of concurrently receiving a composite outcome of high PEEP, high dose of inhaled NO or ventilation in the prone position, suggesting that the “C-type” profile of COVID-19 ARDS patients represents a distinct sub-phenotype of COVID-19 ARDS, irrespective of illness severity, that may be more likely to require advanced therapies to improve oxygenation.

**Point 7:** Lines 23, 32: please avoid or define non-standard abbreviations in the abstract, including “PLIS” and “LUS”
Thank you for this feedback. We have edited the abstract accordingly and made the suggested changes to avoid non-standard abbreviations in the abstract. E.g. line 23 in revised manuscript

**Point 8:** Lines 31, 130: please use appropriate capitalization and subscript for “PaO2/FiO2 ratio”

Thank you for this feedback. We have the made the suggested changes throughout the manuscript to ensure correct notation for PaO2/FiO2 ratio.

**Point 9:** -Line 42: the authors should rephrase “asymptomatic carriage”; could simply say “asymptomatic”

Thank you for this feedback. We have edited accordingly as advised and the sentence has been changed. **See line 43** in the revised manuscript.

**Point 10:** -Lines 44-45: I would argue against the description of COVID-19 as “ambiguous clinical presentation”. Rather, these patients routinely present with symptoms of dyspnea and increased work of breathing. Instead, I would describe how these patients often present similarly but early in their course it can be difficult to differentiate those who will progress to severe disease.

Thank you for this feedback. We have edited accordingly as advised **and the phrase “ambiguous clinical presentation”** has been removed.

**Point 11:** Line 53: use distinct rather than distinctive here
Thank you for this feedback. We have edited the abstract accordingly **and made the recommended change** (see line 52)

**Point 12:** -Line 54: I believe you mean restrictive fluid strategies (rather than liberal), as liberal use of fluids is not a therapeutic intervention in ARDS
Thank you for this comment. We did intentionally refer to a liberal fluid strategy. A secondary analysis of FACTT trial identified heterogeneity of treatment effect in that patients with the hyper-inflammatory sub-phenotype of ARDS seemed to benefit from a liberal fluid strategy, in contrast to the hypo-inflammatory sub-phenotype.

The pertinent citation for this is included here:

Famous KR, Delucchi K, Ware LB et al (2017) Acute respiratory distress syndrome subphenotypes respond differently to randomized fluid management strategy. Am J Respir Crit Care Med 195:331–338. <https://doi.org/10.1164/rccm.201603-0645OC>

**Point 13:** -Line 66: insert comma after COVID-19. Also, this sentence needs to be shorted.
Thank you for this comment. The sentence was edited as suggested. See lines 62-66

**Point 14:** -Line 71: This should be a new sentence (after reference 18).
Thank you for this comment. The sentence was edited as suggested.

**Point 15:** -Line 78: intensive care unit should not be capitalized

Thank you for this comment. The first letter of each word in this phrase was intentionally capitalized as here we defined the abbreviation “ICU” for the rest of the manuscript.

**Point 16:** -Table 1: please reformat as the current formatting of data is confusing. Continuous data (mean ± SD) should be written as such. For example, age (years) should be written as 65.3 ± 11.9 rather than 65.3 (11.9). Non-continuous data should be written, for example for males, as 16 (69.9%) and specified as n (%) rather than n, %.

Thank you for this comment. The table was reformatted as suggested

**Point 17:** -Line 157: POCUS is used as an abbreviation and has not yet been defined.

Thank you for this comment. The phrase POCUS was removed in this instance and was defined in the introduction (line 74).

**REVIEWER #3**

**Point 1:** It was a pleasure for me to read your manuscript and your previous work on the PLIS scanning protocol. I am personally a big fan of this thought process, and in general, I am an advocate for bedside phenotyping of ARDS.

Thank you!

**Point 2:** I suggest the methods to be explained a little bit more in detail in terms of patient selection. I sense that a certain amount of patients admitted to your center were screened, and that only patients that had "enough" scans were considered for the composite outcome. In my mind, it should be explained more clearly who was screened, and hence what were the inclusion and the exclusion criteria (I see ECMO clearly stated).

Thank you for this comment. To clarify – the number of lung ultrasound scans obtained on a patient had no bearing on the data included for analysis. As detailed in the methods, all patient who met the inclusion criteria were included for analysis. This included a patient admitted between the dates of April 1st and September 30th, 2020 with a positive polymerase chain reaction for SARS-COV2 virus who required invasive mechanical ventilation, and were diagnosed with ARDS as set forth in the Berlin criteria. The only exclusion criteria excluded patients meeting this inclusion criteria who during their illness were treated with extracorporeal membrane oxygenation. **This is detailed on lines 89-93.**

**Point 3:** Table 2 should have driving pressure, and tidal volume per kg of IBW among the variables.

Thank you for this comment. Unfortunately, due to the retrospective nature of this research these variables were not consistently recorded in the medical record to access for data collection. We agree that these would be useful for analysis and hopefully can be collected in a future larger prospective research endeavor. We believe that our current findings to be hypothesis generating with enough evidence to pursue this field of inquiry further.

**Point 4:** I like the concept of Figure 3, but if this is a study on 23 patients, I think they should all be presented instead of 10, either as supplemental material or in the main manuscript if physically possible.

Thank you for this feedback. The figure graphically represents the course of ten ventilated patients with COVID-19 ARDS, or whom half survived and half died. This is meant to serve as a visual representation of our study concept and does not represent statistical findings or conclusions. As stated, patients with the most available PLIS data were selected to represent the cohort visually. **A new table has been added to the manuscript including the data on the entire cohort as requested – see supplemental table S2 for comprehensive presentation of study data.**

**Point 5:** The choice for advanced ARDS interventions mentioned in the composite outcome seem to correlate with the C-phenotype more than oxygenation. But it is oxygenation what normally clinically drives these interventions. It makes me wonder whether in your center you include ultrasound evaluation to back up those decisions at rounds. I guess it could make sense in the setting of widespread ultrasound use and proficiency. Also, with this being a retrospective study, I imagine that there is a chance that the arterial blood gases do not always match the scans. These are all aspects that should be covered in the methods, in the discussion and in the conclusion.

Thank you for this comment. Real-time sonographic PLIS scores were not used to guide clinical practice but were collected for isolated research purposes to assess for clinical associations. During the data collection at the beginning of COVID-19 pandemic, the clinical management of the patients was not influenced by the PLIS findings or the presence and size of consolidations. This lack of impact stemmed from the uncertainty regarding how to translate the findings into practical clinical care (as mentioned lines 285-288). In general, nowadays the culture within our center is positive towards the use of point of care ultrasound and critical care echocardiography, however the retrospective nature of this study revealed the b and c patterns or phenotypes long after the study has ended. We wanted to use lung ultrasound to reflect the severity of illness and replace chest X-ray in real time. The association of the b and c and treatment modalities was not obvious in real time - and never used to tailor treatment or guide proning/vasodilator/PEEP therapies. Or analysis reveals this association and calls for future research to better determine how these findings may translate to clinical decisions prospectively.

Regarding the comment on the composite outcome, while oxygenation plays a role in some of these interventions (i.e proning), there are other separate aspects of ARDS pathophysiology that are captured by the composite – mainly minimizing ventilator-induced lung injury. For example, a high PEEP strategy may be used to facilitate lung protective ventilation and minimize driving pressure in the absence of hypoxia, or a clinician may use inhaled nitric oxide if there is evidence of Cor Pulmonale from ARDS. Radiographic subtypes of ARDS have previously been described, stratified into nonfocal and lobar types with different compliance patterns.

We hypothesize similarly that different patterns of lung compliance are associated with C vs B type ultrasonographic patterns separate to patient oxygenation status.

**Point 6:** Finally, I would add a supplementary table about the advanced interventions with the PaO2/FiO2 ratio at the moment when the advanced intervention were started, what was the PLIS score that day, the group B vs C the patients "belonged to" that day, the average daily score from admission to advanced intervention, and the average daily score from intubation to advanced intervention, if data is available.

Thank you for this comment. While our group has previously described and published on the utility of the PLIS score for clinical care, the purpose of this research endeavor focused on stratifying patients by “B” and “C” phenotype by the presence or absense of ultrasonographic consolidations. Thus the final comprehensive PLIS score was not a core component of our analysis, though we did report the median PLIS scores between cohorts in Table 2.

With this feedback, we have created a new table, that includes some of the available data as requested. See table S2 for further comprehensive summary details of the cohort.
