# Supplementary material for: Lung ultrasound is associated with distinct clinical phenotypes in COVID-19 ARDS: A retrospective observational study
Source: PLoS One. 2024 Jun 3;19(6):e0304508. doi: 10.1371/journal.pone.0304508 (PMC11146726; doi:10.1371/journal.pone.0304508)
Supplement: Attachment — Submitted filename: PLOS One- Response to Reviewers - March2024.docx [file pone.0304508.s007.docx]

**Dear Editor in chief**,

Thank you for giving us the opportunity to submit a revised draft of the manuscript “Lung ultrasound is associated with distinct clinical phenotypes in COVID-19 ARDS: A retrospective observational study” to PLOS ONE for consideration for publication.

We appreciate the time and effort you and the reviewers dedicated to providing feedback on our manuscript. We have incorporated most of the suggestions made by the reviewers. Those changes are highlighted within the manuscript.

Please see below **in red** for a point-by-point response to the reviewers’ comments and concerns.

All page numbers refer to the revised manuscript file, entitled “manuscript” as directed.

**REVIEWER #1**

***Point 1*:** “*The authors propose that patients may exhibit various phenotypes throughout their clinical course. However, the analysis of outcomes associated with phenotypes C and B appears to represent two distinct populations. It remains unclear at which point in the patients' clinical history the lung ultrasound (LUS) was conducted and when a patient was classified as having phenotype C or B.”*

Thank you for this feedback. Lung ultrasound (LUS) scans were performed bedside during morning rounds as part of routine clinical assessment as an extension of the physical exam, as well as during specific clinical deterioration events, and findings were formally documented in the medical record. A scan was determined as a B-type or a C-type based on the size of lung consolidation measured In the mid-posterior axillary line. Each patient could shift between the “B type” and “C type” phenotypes throughout their course, according to the findings from the specific scan conducted that day. This is detailed in the manuscript in **lines 95-102 and 110-111.**

We agree that our research suggests that phenotype C may represent a specific population that can potentially receive targeted therapeutic interventions.

**Point 2:** “*Although the group refers to a previous ultrasound score, providing a more detailed explanation of the ultrasound evaluation would enhance clarity. Specifically, it would be helpful to elaborate on which part of the thorax was assessed. For instance, determining whether the dorsal region, where larger consolidations are typically found, can be reliably assessed by ultrasound, especially in patients in the prone position, would be valuable. This aspect is crucial to the methodology and limitations, as an inability to evaluate a certain thoracic region via ultrasound may lead to a potential underestimation of the phenotype*.”

Thank you for this feedback. We agree that the location of ultrasound image acquisition is essential for appropriate clinical application. A detailed explanation of the previously published PLIS ultrasound score was included in the supplemental materials (See Fig. S2 and Table S1). **In this revised manuscript in response to the reviewer’s comments, parts of Fig S1 and Fig S2 have been combined and added to the body of the manuscript and have been re-labeled as Fig 1 – Lines 99-100 (Fig 1 legend – Lines 105-109)**

In additional response to this specific feedback given here in point 2, we have added the following to elaborate on the “C” component of the PLIS, pertinent to our study – **see lines 99-100.**

**“All ultrasound images evaluating for consolidations were obtained in the mid-posterior axillary line above the diaphragm (Fig.1, designated zone 2)”.**

The broader details of the PLIS were included in the supplement to shorten the introduction, which has already been referenced as being lengthy. Our current study focuses exclusively on the “C” component of the PLIS and not on the broader previously published PLIS score which is included primarily for reference.

**Point 3: “***The introduction is lengthy; it would benefit from being more concise. To achieve this, consider avoiding the repetition of studies and concepts already discussed in the subsequent sections.”*

Thank you for this feedback. To shorten the introduction and avoid repetition, several edits have been made and content has been removed and/or abbreviated. The revised introduction is significantly shortened. **The new version of the introduction is 421, previously 524 words.**

**REVIEWER #3**

**Point 1:** “*Thank you for resubmitting. Thank you for clarifying the inclusion/exclusion criteria in your dataset. I agree that oxygenation should be only one small part of what triggers interventions, which is why I was hoping for more data regarding protective ventilation. But I appreciate your clarifications about the method and how hypothesis were generated.
US can complement CXR to quickly photograph patients that have less homogeneously ventilated lungs, shunt more, and trigger specific treatments. Please, keep up: an ultrasound-trained generation of intensivists is expected to multiply the number of patients and observations, correlate with other imaging techniques and patient's respiratory mechanics.”*

**Thank you for this feedback! We appreciate the kind words. We fully agree** – we believe our work can help lay the foundation for the role of POCUS to guide therapies in ARDS.
